# Cell Synchronization Enhances Nuclear Transformation and Genome Editing *via* Cas9 Enabling Homologous Recombination in *Chlamydomonas reinhardtii*

**DOI:** 10.1021/acssynbio.0c00390

**Published:** 2020-09-11

**Authors:** Max Angstenberger, Francesco de Signori, Valeria Vecchi, Luca Dall’Osto, Roberto Bassi

**Affiliations:** † Department of Biotechnology, University of Verona, Cà Vignal 1, Strada le Grazie 15, 31734 Verona, Italy

**Keywords:** *Chlamydomonas reinhardtii*, genome editing, CRISPR/Cas9, nonhomologous end joining, homologous recombination, *cpftsy*

## Abstract

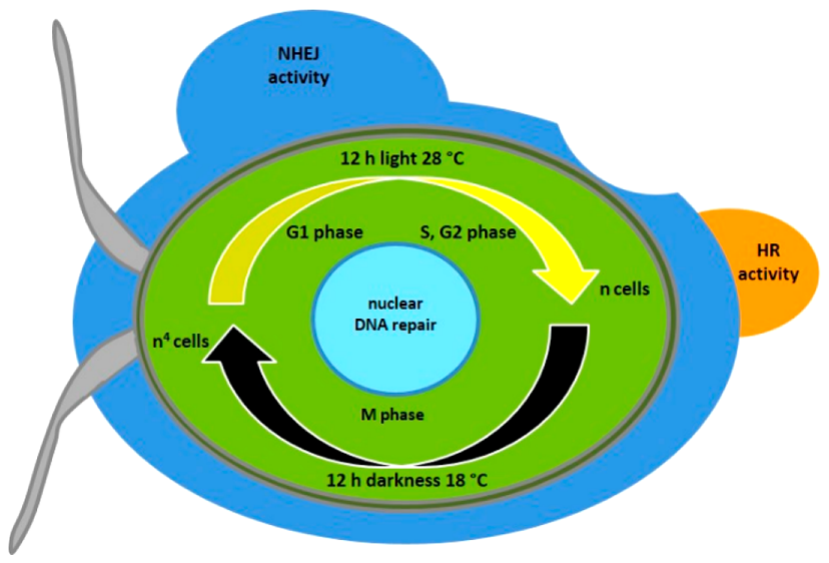

In *Chlamydomonas reinhardtii*, the model organism for eukaryotic green algae and plants, the processes of nuclear transformation and genome editing in particular are still marked by a low level of efficiency, and so intensive work is required in order to create and identify mutants for the investigation of basic physiological processes, as well as the implementation of biotechnological applications. In this work, we show that cell synchronization during the stages of the cell cycle, obtained from long-term cultivation under specific growth conditions, greatly enhances the efficiency of transformation and allows the identification of DNA repair mechanisms that occur preferentially at different stages of the cell cycle. We demonstrate that the transformation of synchronized cells at different times was differentially associated with nonhomologous end joining (NHEJ) and/or homologous recombination (HR), and makes it possible to knock-in specific foreign DNA at the genomic nuclear location desired by exploiting HR. This optimization greatly reduces the overall complexity of the genome editing procedure and creates new opportunities for altering genes and their products.

As the model organism for eukaryotic green algae and plants, *Chlamydomonas reinhardtii* has been widely studied over recent decades, as summarized in ref (1). Its fully sequenced^2^ haploid genome offers great benefits in establishing techniques for nuclear transformation^3^ and electroporation,^4^ as well as genome editing techniques such as the CRISPR/Cas9 system.^5,6^ Special strains that are even easier to transform are also available, for example, the CW15 strain with a reduced cell wall.^7^ Nevertheless, transformation, and specific gene targeting techniques in particular, still need to be further optimized in order to obtain an efficient platform for creating mutants that can be applied to other algae and plant species.

The recent confirmation of the transformation of *C. reinhardtii* using ribonucleoproteins^6,8^ of Cas9 and coupled single-guide RNA (sgRNA) opened the way for the production of gene knockout mutants and, in some cases, the knock-in of foreign DNA delivered in specific sequences (compare Figure 1).

**Figure 1:**
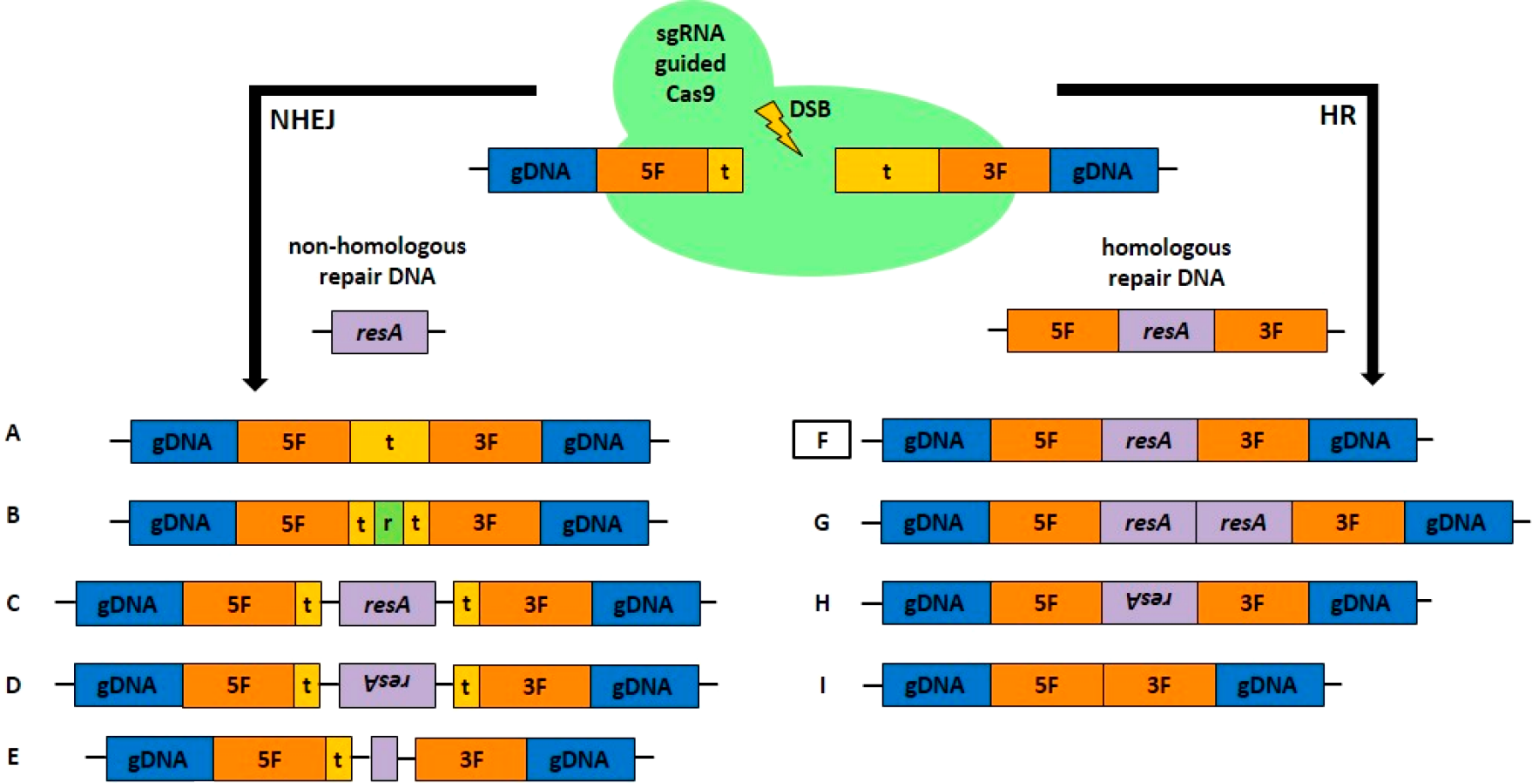
Schematic overview of single guide RNA (sgRNA), guided Cas9 introduced DNA double-strand breaks (DSB), and possible DNA repair pathways in eukaryotes. Repair mechanisms include nonhomologous end joining (NHEJ) and homologous recombination (HR), the possible outcomes of which depend on the provision of DNA repair and the respective active repair mechanism. In the uppermost part, the selected exemplary nuclear (gDNA) target sequence (t) and 5′ and 3′ flanking regions (5F, 3F) are depicted. Using sgRNA-guided Cas9, a DSB can be introduced in the target region, but the process is not entirely predictable. As long as a nonhomologous repair template confers antibiotic resistance (*resA* cassette), an active NHEJ pathway can lead to several results: (A) target sequence repair with error-prone sequence modifications, (B) insertion of random (r) sequences, (C) insertion of DNA clockwise, and anticlockwise in (D), and (E) insertion of truncated repair DNA. As a consequence, there are likely to be remnants of the chosen target sequence. In contrast, the provision of homologous repair DNA containing the flanking regions can lead to different results in an active HR pathway: the most desirable outcome is shown in (F), that is, the sequence-specific replacement of the target sequence with *resA*. Other options are multiple insertions of *resA* (G), inverted insertions (H) and deletions (I).

The latter was recently confirmed^9^ as enabling unspecific knock-ins *via* nonhomologous end-joining (NHEJ), which displayed the disadvantage of sequence changes often observed during DNA integration (Figure 1C,E) which, together with low transformation efficiency, makes identification of specific genotypes problematic and requiring extensive sequencing procedures. Therefore, specific knock-ins caused by HR (Figure 1F–I), with a high probability of complete and sequence-specific DNA integration, display great potential in altering gene structures and therefore altering protein sequences, to implement protein tagging systems,^10^ for example, or to replace genes with copies containing single point mutations. Significantly, such specific knock-ins created through the HR pathway (Figure 1F), that is, replacing a target sequence using homologous flanking regions, only appear naturally at very low frequencies in *C. reinhardtii*;^11−14^ instead, random DNA integration is dominant and mediated by NHEJ.^15^ Inducing DNA double-strand breaks (DSB) using the Cas9 nuclease at specific sequences and providing homologous repair templates were shown to increase the probability of specific knock-ins in various species.^16,17^ HR activity is often suppressed by the NHEJ pathway in eukaryotes^18^ and is only present in certain stages of the eukaryotic cell cycle,^19^ that is, in the late DNA synthesis phase (S phase) and early mitotic phase (M phase). In addition, HR activity is well-known from the recombination of parental DNA in meiosis.

Various attempts to improve HR efficiency in microalgae as well as in other species have been reported in recent years, including, for example, by interfering with the NHEJ-specific DNA-Ligase IV,^20,21^ inducing DSBs, or taking advantage of synchronized cells in order to access HR specific stages of the cell cycle for transformation, for example, using a zinc-finger nuclease.^16^ However, the level of HR efficiency so far obtained is still lower than that required to make these procedures part of the daily routine of the genetic modification of *C. reinhardtii*. Therefore, a more profound synchronization of cells in a culture during the eukaryotic cell cycle stages at the same time display a promising advantage to optimize genome editing in *C. r.* by exploiting the differentially present DNA integration mechanisms during the cell cycle and though could serve as a model system for similar strategies in other algae species. It was our hypothesis that a relaxed chromatin structure, present during the interphase in all synchronized cells of *C. r.* before entering the mitotic prophase with associated chromatin condensation,^22^ could enhance DNA integration. Moreover, such chromatin state is expected to offer a more accessible target for the Cas9 nuclease in order to introduce a DSB for repair by NHEJ or HR. The latter mechanism, which could be displayed at a specific cell cycle stage, could lead to a specific knock-in of a transformed DNA molecule containing homologous flanking regions to a defined target site (Figure 1F).

Cell synchronization depends on light and temperature (12 h of light at 28 °C and 12 h of darkness at 18 °C) and was shown to be obtained during short-term cultivation,^23^ leading to one cell division per night (M phase) and cell growth during the day (interphase) in *C. reinhardtii*. Moreover, depending on specific growth conditions, *C. r.* can even undergo multiple cell divisions consisting of S and M phases^24^ including chromatin decondensation.^25^ Further, cells enter the interphase again starting with the G1 phase that can last for several hours^26^ and includes an extended decondensed chromatin structure. Between S and M phase, eukaryotes generally exhibit a second growth phase G2, that is either not present or difficult to detect in *C. r.*(27) In order to reach optimal cell synchronization, long-term cultivation in such conditions^23^ was therefore chosen to investigate the possibility of maximizing genome editing (GE) efficiency by accessing each stage of the cell cycle for transformation and so define specific properties for DNA integration depending on NHEJ which is dominant in the G1 phase in eukaryotes^28^ and/or HR, that is present in S and early M phase. Determining the transformation efficiency (obtained clones/used ng DNA for transformation/transformed cells) at certain stages of the cell cycle, using either nonhomologous or homologous transformation constructs, should therefore provide information about the ongoing DNA repair mechanisms (NHEJ/HR), while target sequence analysis could confirm the occurrence of specific knock-ins.

For the prompt detection of transformation events, the gene *cpftsy*(8,9,29) was chosen as the target. The associated protein is involved in the assembly of the light-harvesting system in the thylakoid membranes. When absent due to a knockout, mutants display a pale green phenotype that is easily detected by the naked eye on plates during growth after transformation. This approach made it possible for the knockout frequency to be quickly determined by counting the pale green colonies as compared to the native green ones, created by random mutations. Interestingly, the knockout frequency of *C. r. cpftsy* was very low in earlier experiments (0.5–1%) compared to knockouts of other target genes, for example, the chlorophyllide-a oxygenase (*CAO*, ∼20%, data not shown), which also leads to a pale phenotype, though less pronounced. Nevertheless, analyzing the low knockout frequency of the *cpftsy* gene offers the advantage of showing the full effect of an optimized GE frequency, avoiding saturation. To enable specific recombination events by HR at the *cpftsy* locus, a construct with 2 kb flanking regions surrounding the sgRNA guided target sequence was created. Large flanking regions were previously shown to enhance HR events in *C. reinhardtii*(30) and in other microalgae,^21^ increasing the probability of such events.

## Results and Discussion

### *C. r.* CW15 Cell Synchronization under Long-Term Cultivation

**Figure 2:**
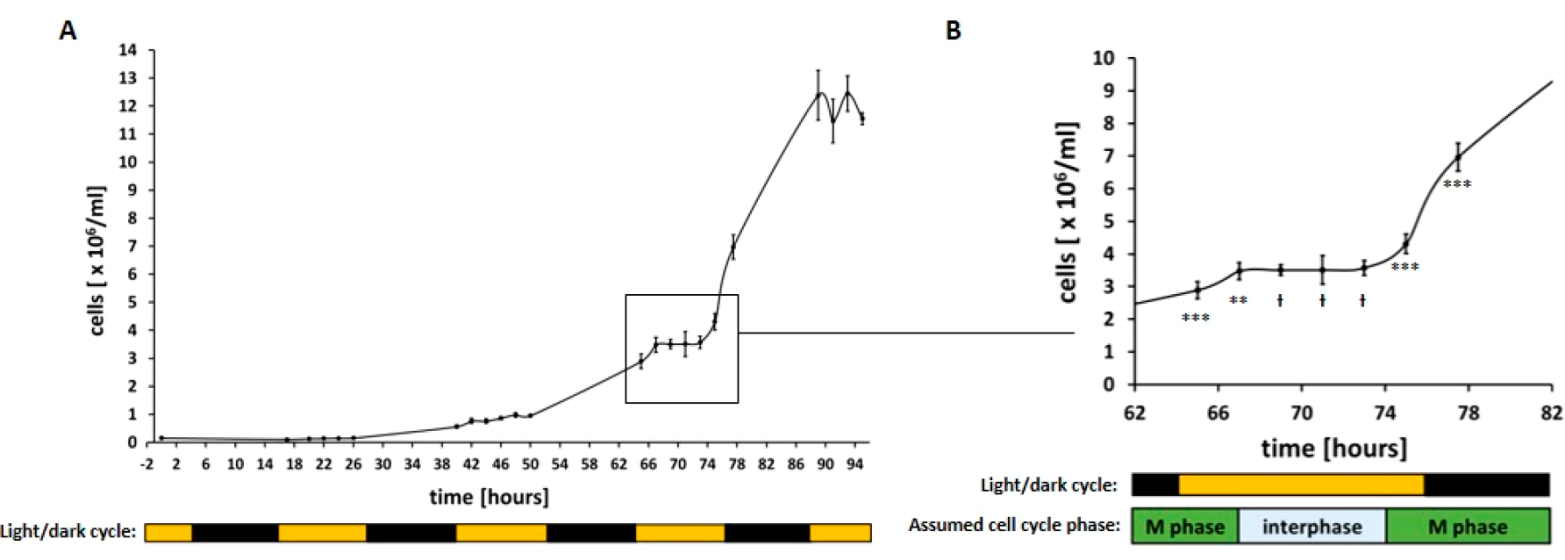
Growth curves of synchronized *C. r.* CW15 cultures. The number of cells of *C. r.* CW15 grown at 28 °C during the light phase (12 h, 200 μmol photons m^–2^ s^–1^) and at 18 °C (12 h) during the dark phase (as indicated by bars) was determined using a cell counter after 2 weeks of preculturing under the same conditions. Each data point represents an average of three technical replicates that were each counted twice. (A) Overview of cell number during growth for 4 days. (B) Enlarged view of the curve after 3 days of growth. Assumed cell cycle phases are shown as bars. A one-sided *t* test was performed, and the statistical confidence level is shown in relation to the previous data point as two asterisks for *p* < 0.01, three asterisks for *p* < 0.001, while a hash represents insignificant values of *p* > 0.05.

As reported by ref (23), cell synchronization of *C. r.* can be achieved by applying a temperature of 28 °C during the light phase (resulting in cell growth) and 18 °C in the dark phase (leading to an exact doubling of cell numbers). We observed the growth behavior of CW15 in similar light-dark and warm-cool cycles, as carried out by ref (23), in a multicultivator system that measured the optical density over several days (not shown). Interestingly, under these growth conditions (12 h light at 28 °C and 12 h darkness at 18 °C), we observed a change in growth behavior during prolonged cultivation, leading to a higher daily cell number than expected, if two daughter cells were being generated by each mother cell. In order to further characterize such growth behavior, a batch culture of CW15 was maintained under these conditions and after 3 to 4 days of growth, cells were used to inoculate a new culture, in order to achieve a long-term adaptation (2 weeks of preculturing with ongoing cultivation). Following a subsequent inoculation, the cell number was determined continuously using a cell counter (Figure 2A, B).

As expected, a pretty constant cell number was measured during the light phases (Figure 2A), but a significant cell number increase was observed during each dark phase. In addition to the previous report^23^ in which the cultivation was short-term, we saw higher cell division rates after long-term cultivation with approximately 2 cell divisions during each dark phase, as previously described by^24^ to be possible in *C. r*.

On the third day after inoculation in particular (Figure 2B), the expected synchronization of the culture could be observed. After 3 h of light, cell division stopped and the cell number remained constant for about 6 h before cell division resumed. In the following 4.5 h, the cell number doubled, from 3.57 × 10^6^ cells/mL to 6.97 × 10^6^ cells/mL (a factor of 1.95), implying synchronized cell division was underway in the culture. A second cell division occurred during this dark phase, resulting in 12.4 × 10^6^ cells/mL the next day (a factor of 1.78). On the basis of general knowledge of the eukaryotic cell cycle, we could assume that the two major phases (interphase and M phase, Figure 2B) were underway in the vast majority of the CW15 cells at different points of time during this cultivation procedure. Due to the occurrence of two subsequent cell divisions in the dark phase, a second interphase (though shorter) occurred, as described by ref (31). This shorter interphase was not examined further and so is not displayed in the M phase in Figure 2B.

### Determination of Transformation Efficiency in Synchronized *C. r.* CW15 Cultures

**Figure 3:**
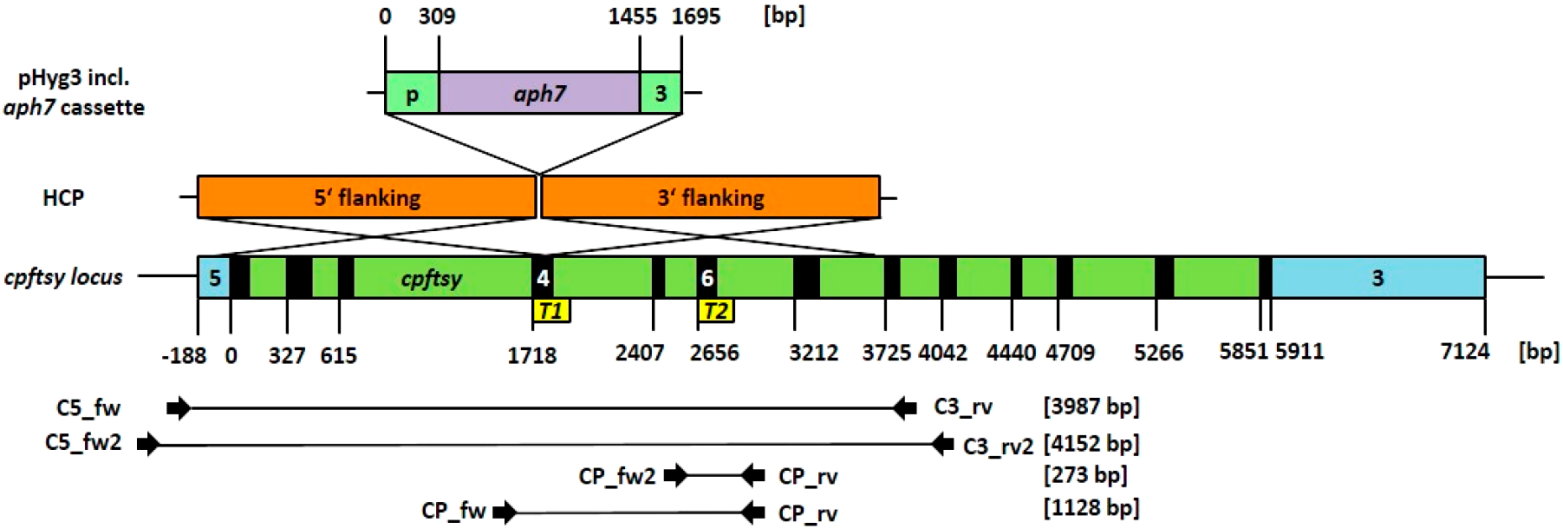
Overview of used transformation constructs and the target locus of *cpftsy* depicted as true-to-scale schematic representations of different elements. (Top) The standard transformation vector pHyg3 for *C. r.* contains the antibiotic resistance cassette, consisting of the promoter (p) of the β-Tubulin gene, the hygromycin resistance gene *aph7* and the 3′ UTR (3) of the RubisCO small subunit 2 gene. (Middle) The knock-in construct HCP (homologous recombination for *cpftsy*) targeting the *cpftsy* locus was created by cloning the *aph7* cassette of pHyg3 in between two homologous flanking regions (5′ and 3′ flanking, surrounding a first target sequence T1) into the bluescript vector KS-. (Bottom) The *cpftsy* locus included the 5′ UTR (5), coding exons depicted in black and the 3′ UTR (3). Both target sequences used for creating sgRNA (T1, T2) are shown, as well as the primer binding sites and the sizes of the expected amplification products.

Using CW15 synchronized cultures made it possible to investigate the effect on transformation efficiency and the occurrence of different DNA repair mechanisms (NHEJ/HR) by using a nonhomologous (pHyg3, Figure 3) or homologous linearized transformation construct (HCP, containing homologous flanking regions of *cpftsy*, Figure 3) at different time points during cultivation. Random DNA integration by NHEJ was therefore expected to lead to hygromycin-resistant, normal green colonies, whereas HR-mediated recombination events at *cpftsy* should generate pale green clones.

**Figure 4:**
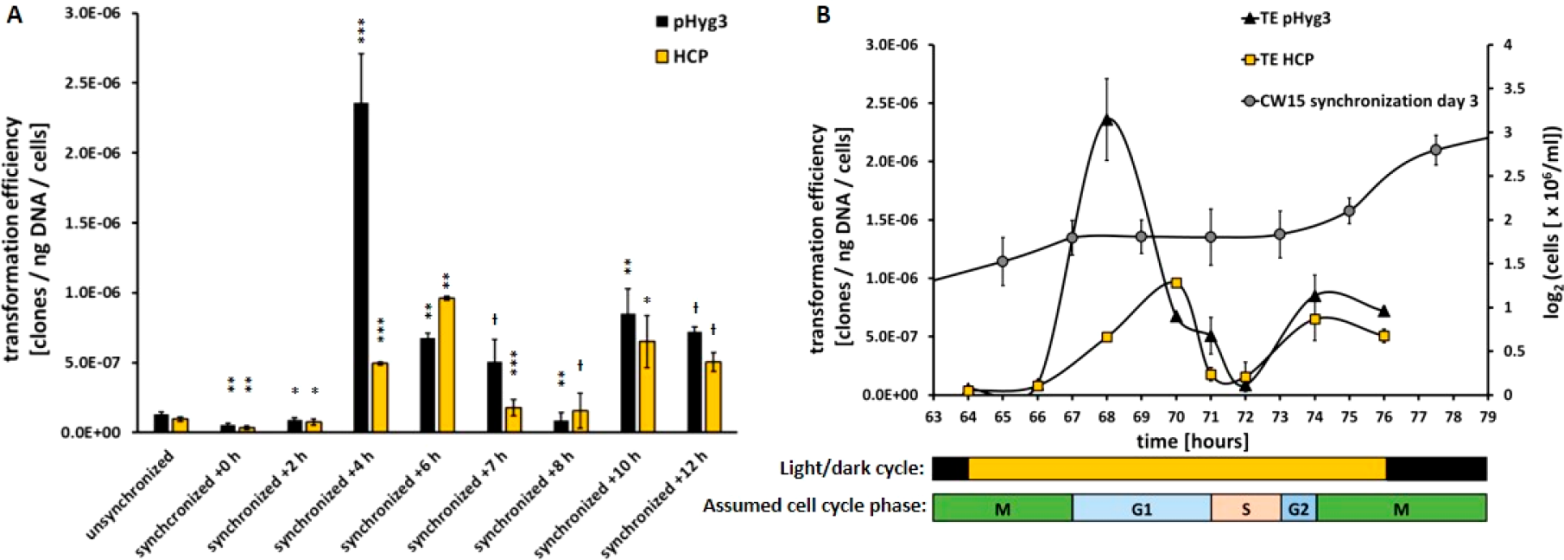
Analysis of transformation efficiency of unsynchronized and synchronized cell cultures of *C. r.* CW15. (A) TE was measured at different time points (h, hours after illumination start) after 3 days of cultivation under synchronizing conditions. Each data point shows mean ± SD, *n* = 3. Transformations by linearized construct pHyg3 (nonhomologous) are depicted in black, and those by linearized construct HCP (homologue to *cpftsy*) are shown in yellow. A one-sided *t* test was performed and the statistical confidence level is shown in relation to the previous related data point as one asterisk for *p* < 0.05, two asterisks for *p* < 0.01, three asterisks for *p* < 0.001, while a hash represents insignificant values of *p* > 0.05. (B) TE data is displayed in comparison with the corresponding cell number (shown as log_2_, gray circles) on the third day of growth under synchronizing conditions (compare Figure 2B). Below, the illumination cycle is depicted as bars, as well as the assumed cell cycle phases.

First, we defined an optimized transformation protocol aimed at minimizing false positive clones and simplifying the procedure based on.^4^ The final optimized protocol is described in detail in the Methods section (see also Table S1) and includes the reduction of materials, working time and a more stringent selection using a higher hygromycin concentration. Once optimized conditions on synchronized CW15 had been established, the analysis of transformations at different time points on the third day of cultivation with respective constructs (Figure 4A) displayed a strong enhancement of the transformation efficiency (TE).

Compared to the TE of unsynchronized cells, the TE using synchronized cells and pHyg3 remained rather low during the first hours of illumination (around 10^–7^ clones/ng DNA/cells), but was drastically enhanced to a maximum at +4 h of illumination (+1800%). In the following hours, TE decreased to a pronounced minimum at +8 h, but increased again at the end of illumination period to around 5 × 10^–7^ clones/ng DNA/cells. TE using HCP displayed a similar pattern, but interestingly reached a maximum at +6 h (∼10^–6^ clones/ng DNA/cells), although less pronounced compared to that for pHyg3. No pale colonies could be found after all those transformations, confirming the aforementioned inefficiency of the HR pathway in *C. r*. Nevertheless, the different maxima of TE using pHyg3 and HCP point to the recognition by the cell of homologous foreign DNA, which cannot be used for HR-mediated recombination in this physiological state, probably due to missing DNA DSBs in the target sequence *cpftsy* and/or an inactive HR pathway caused by a lack of necessary proteins.

By combining the TE values of synchronized CW15 cells with the cell number on day 3 after inoculation (Figure 4B), it was possible to associate the defined cell cycle stages based on TE tendency. As with the G1 phase, NHEJ is the dominant DNA repair pathway, reflected in the maximal TE using pHyg3 at +4 h of illumination. The following minimum of TE at +8 h can be explained by cells entering the S phase, in which DNA integration must be avoided in order to protect the karyome from the random rearrangement of the newly synthesized DNA. Nevertheless, the possibility that HR could be active at this stage was further investigated. Before entering the M phase, eukaryotic cells undergo a shorter G2 phase, although not described by the literature^23,27,31^ for *C. r.*, which we associated with around +9 h after illumination. Cells then re-enter the M phase at around +10 h of light and begin to divide. HR activity was therefore expected to be present during the last hours of illumination in order to enable sequence-specific DNA repair to take place before cell division. These results strongly suggest the need for the Cas9 nuclease to introduce DNA DSBs enabling recombination events.

### Genome Editing of Synchronized CW15 Using the Cas9 Nuclease

The Cas9 nuclease was expressed in *E. coli* and purified using Ni-affinity chromatography. The presence of recombinant enzyme was evaluated by SDS-PAGE in the eluted fractions (Figure S1A), which showed a high degree of purity. Another critical feature for a high activity was the removal of bacterial nucleic acids, achieved by EDTA incubation and subsequent washing to remove the chelating agent. The concentrated Cas9 preparation (Figure S1B) was further evaluated by immunodecoration analysis using an α-His tag antibody (Figure S1C). An *in vitro* assay proved the capability of restricting the defined target DNA sequence of *cpftsy* (Figure S1D) in the presence of the respective sgRNA (T1, T2). Since both target sequences are close to the end of the DNA molecule (Figure 3), little change in size occurred, but it nevertheless proved to be a functional Cas9 nuclease.

Interestingly, while testing different target sequences as sgRNA for *cpftsy* in transformations (not shown), we saw that not all events led to the creation of a pale phenotype, suggesting the inefficiency of *cpftsy* related sequences in genome editing. The defined target sequence 1 (T1,^8^Figure 3) was therefore maintained and an additional target sequence (T2, Figure 3) was chosen. Both sgRNAs led to the creation of pale mutants and so were used together for genome editing experiments. Although T2 is also present in HCP (Figure 3), a restriction of the homologous template was not thought to interfere with putative HR activity, since about half of the homologous flanking region would still be present and because there was no incubation of Cas9 ribonucleoproteins together with HCP at activating temperatures prior to transformation.

**Figure 5:**
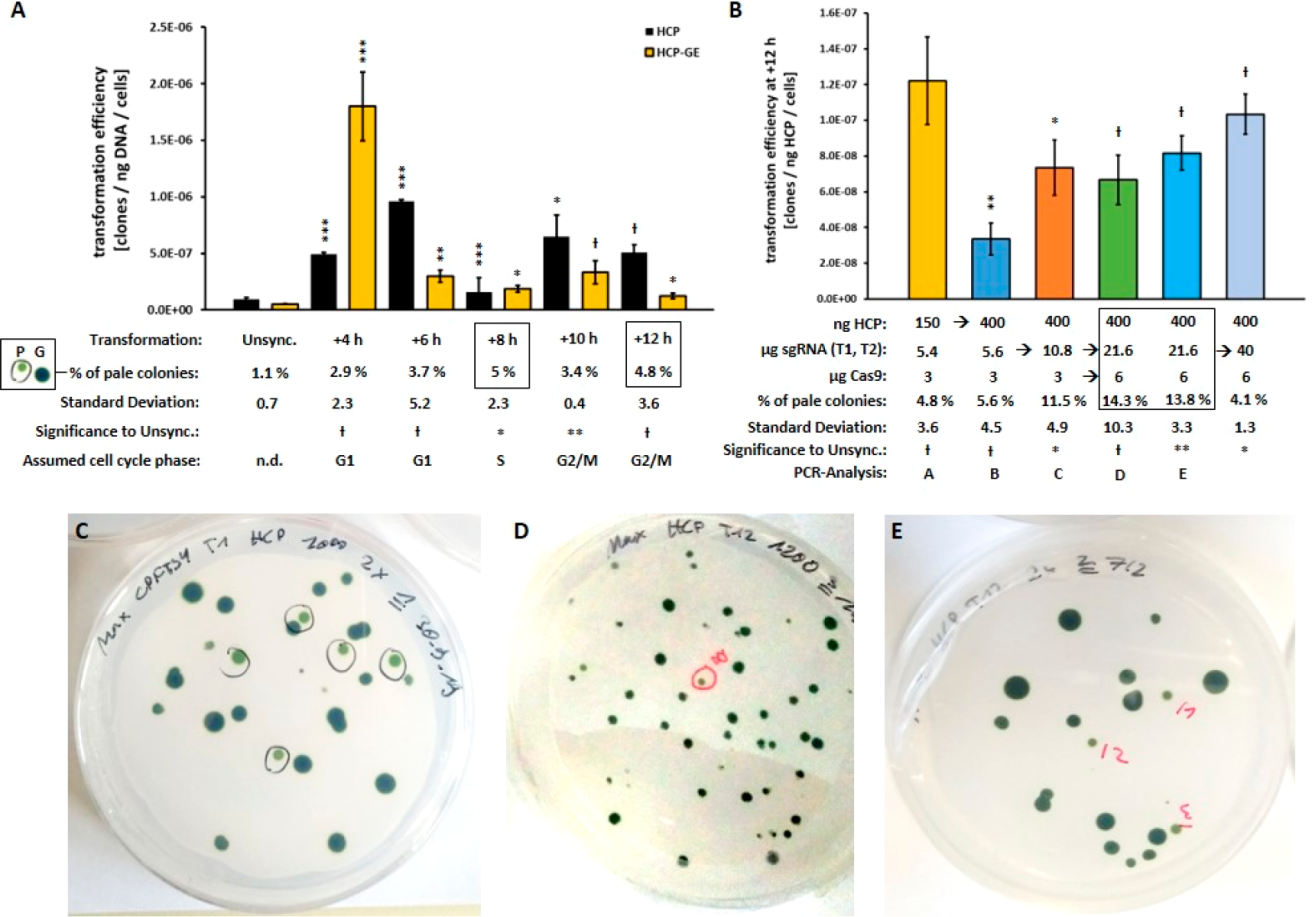
Transformation efficiency of unsynchronized and synchronized cell cultures of *C. r.* CW15: Effect of ribonucleoprotein Cas9 on the transformation efficiency. (A) TE were determined for unsynchronized (unsync.) and synchronized cell cultures of *C. r.* CW15, using the linearized *cpftsy* homologous HCP construct only (black bars) or together with ribonucleoproteins (yellow bars, HCP-GE containing Cas9, sgRNAs T1 and T2) at different time points after the start of illumination. Data is shown as mean ± SD, *n* = 3. Below, the determined percentage of the total pale green colonies obtained for HCP-GE (P, of green colonies G) is given: maximal values are surrounded by black boxes. A one-sided *t* test was performed, and the statistical confidence level is shown in relation to the data of unsynchronized cells (unsync.) as one asterisk for *p* < 0.05, two asterisks for *p* < 0.01, three asterisks for *p* < 0.001, with a hash representing insignificant values of *p* > 0.05. This was calculated in the same way as (B). Assumed cell cycle phases are also shown below. (B) Transformation efficiencies of further variations of HCP-GE trials at time point +12 h are shown as mean ± SD, *n* = 3. The amounts used of HCP, sgRNAs T1, T2, and Cas9, as well as the percentage of pale colonies and the numbers for PCR analysis, are given below. The determined optimal ratio of components is marked by a black box. Representative pictures of transformation results, *i.e.*, pale mutants (marked black and red) *vs* normal green mutants are shown as a general example in (C), of transformation using HCP at +4 h (D, compare A), as well as of transformation D at +12 h (E, compare B).

When transforming synchronized CW15 at different time points using Cas9, pale green mutants were obtained in all cases at higher percentages than for unsynchronized cultures (Figure 5A,C–E), underlining once again the advantage of synchronization. Interestingly, TE increased only at +4 h when using HCP together with Cas9 ribonucleoproteins (HCP-GE) compared to the use of HCP alone, underscoring the dominance of the NHEJ pathway in leading to random mutants. In all other cases, TE decreased or was unchanged when additionally using Cas9, pointing to more specific DNA repair, that is, HR.

The maximal efficiencies for creating pale mutants (around 5%) occurred at +8 h and +12 h of illumination, which were associated with the S phase and the G2/M phase respectively, since HR activity has a higher efficiency for functional knockouts and was assumed to be present at these time points. This hypothesis was further supported by the rather low percentage of pale mutants at time point +4 h, which was attributed to the G1 phase with high NHEJ activity. Since TE at +8 h (S phase) was very low (Figure 4B), time point +12 h was chosen for further optimization experiments to test different amounts of the components used (Figure 5B) due to quite a high TE and a high percentage of pale mutants accompanied by the expected HR activity.

Interestingly, the provision of more plasmid DNA (150 to 400 ng HCP) decreased the TE but resulted in a similar percentage of pale mutants, confirming the recognition of the homologous template and, moreover, that the introduction of the DSB was the limiting step. This conclusion was further confirmed when using double the amount of sgRNA (5.4 μg to 10.8 μg) because the TE increased again (+100%) and the percentage of pale mutants was more than doubled (factor 2.1). A further increase of sgRNA (21.6 μg) and Cas9 (6 μg) resulted in the highest percentage of pale mutants observed (14.3% and 13.8%), with an average of 14%. A further increase of sgRNA to 40 μg decreased the yield of pale green clones, which confirms the optimal ratio previously determined. Overall, the efficiency of creating pale mutants could be increased by about 1300% by switching from unsynchronized cells (1.1% of pale green clones) to synchronized cells at +12 h (14%). In principle, this could be optimized even further by using different combinations of components.

### Determination of HR-Mediated Knock-Ins in Pale Mutants

**Figure 6:**
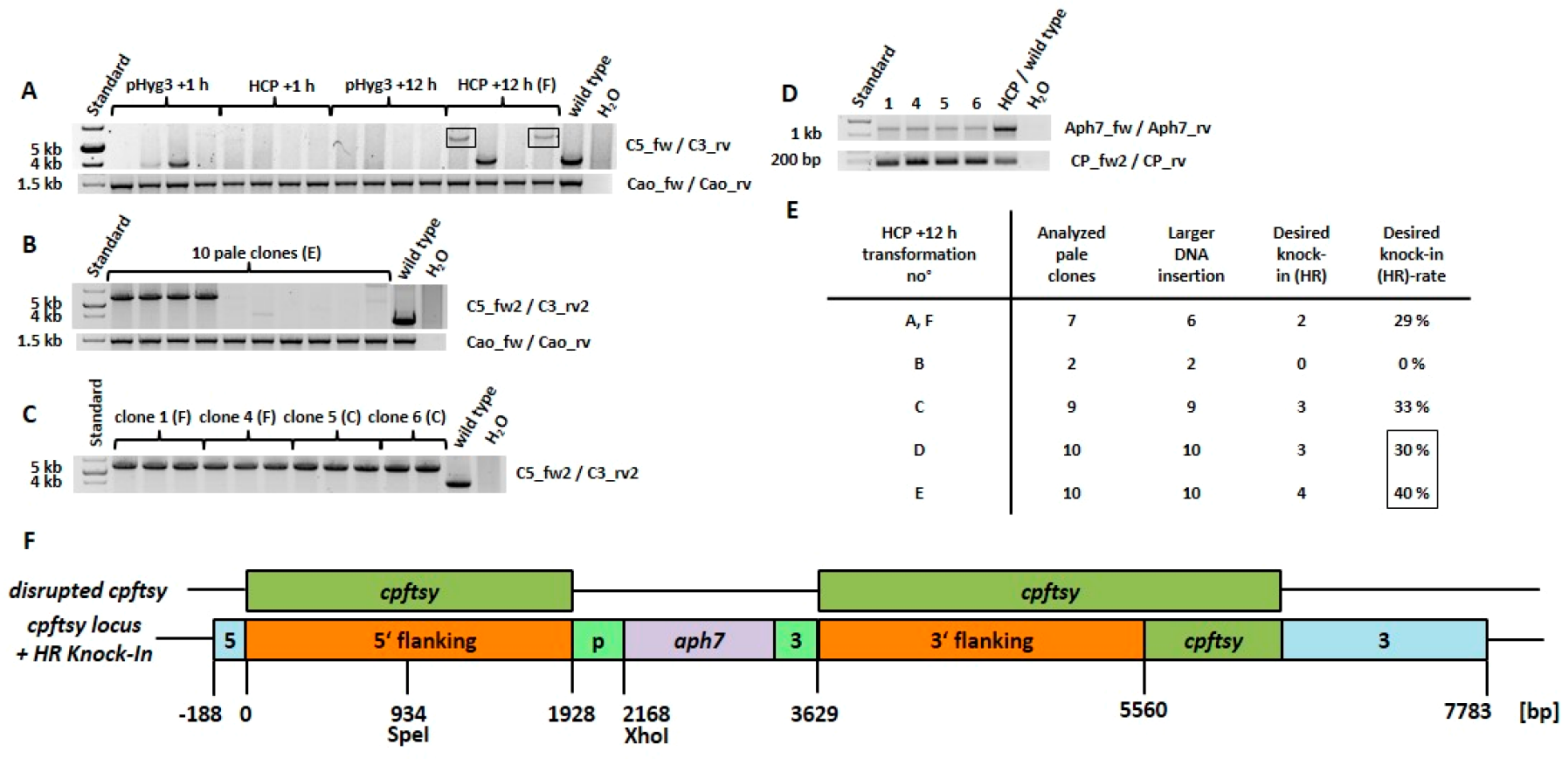
Target sequence analysis of selected pale mutants of *C. r.* CW15 from different transformation approaches. (A) PCR analysis carried out by the flanking region surrounding primers (C5_fw/C3_rv) for locus *cpftsy*. Four pale mutants for each construct (pHyg3, HCP) at two different time points (+1 h, + 12 h) were analyzed together with the wild type; the amplicons expected after HR-mediated knock-in of about 5.8 kb are surrounded by black boxes. (B) Similar PCR analysis were carried out with optimized primers (C5_fw2, C3_rv2) for 10 pale clones from transformation E (see Figure 5B). As positive controls for each cell line, additional PCRs were performed using primers Cao_fw and Cao_rv and are shown in the lower part of the A–B panels. (C) Selected pale mutants from transformations C, F at +12 h (see Figure 5B) were used for the amplification of sufficient amounts of recombinant knock-in fragments of *cpftsy* for purification and further analysis, as shown in (D), that is, Nested-PCRs were performed using the purified fragments as template and primers Aph7_fw/Aph7_rv for detection of the inserted *aph7* gene, in addition to primers CP_fw2/Cp_rv for detection of the target sequence T2 of *cpftsy* as a control. (E) Summary of results of additional PCR analysis performed on pale clones from corresponding transformations of *C. r.* CW15 at +12 h after the start of illumination (see also Figure 5B), showing the numbers of analyzed pale clones, the larger DNA insertions in *cpftsy* detected, the desired HR-mediated knock-ins (flanking-based target sequence replacement) and its occurrence rate. (F) Schematic representation of knock-ins of the *aph7* cassette into *cpftsy* detected, based on HR-mediated recombination, which was confirmed by sequencing the corresponding XhoI and SpeI restricted amplicons (cloned into bluescript vector KS-) from clones 1F and 4F (see Figure S2). The disrupted genomic sequence of *cpftsy* is depicted at the top, showing the occurrence of the gene disruption that leads to the pale phenotype. For the numbering, compare with Figure 5B, except for transformation F, which is not shown in detail.

A major advantage of knock-ins caused by HR is the easy detection of functional knockout mutants by means of PCR screening. Indeed, large insertions can be easily detected and a functional knockout is the highly probable consequence. Since NHEJ-mediated gene disruption in most cases results in only a few base changes,^32^ sequencing of the corresponding target sequence products is required and the function of the knockout has to be determined by other means, for example, by the analysis of the protein content. In order to analyze pale mutants obtained from different time points (+1 h and +12 h of illumination, Figure 6A), PCR was performed using primers for *cpftsy* that bind to the exterior of the flanking regions (Figure 3) in order to avoid false positive PCR artifacts.^33^ Interestingly, the native PCR amplicon could not be detected in most clones from synchronized cultures, indicating a relatively high knock-in frequency (either unspecific, by NHEJ, or specific, by HR) of larger DNA molecules (for example, plasmid) into the *cpftsy* target itself. Clones derived from transformation at +1 h were devoid of the expected HR-mediated recombination product (target sequence replacement) of about 5.8 kb, as had been expected due to the inactive HR pathway. On the other hand, two out of four clones from transformation at +12 h did show the 5.8 kb recombination product, so supporting the presence of HR in the assumed G2/M phase. The total occurrence of HR-mediated recombination events could be even higher at this time point or at others, since there are different outcomes (Figure 1F–I) from recombination-like events, for example, multiple insertions and deletions.^21^ Interestingly, the sought-after knock-ins of HCP into *cpftsy* could not be identified at any time point other than +12 h (tested for +1, +2, +4, +6, +8, and +10 h with about 40 pale clones in total, data not shown).

Pale clones derived from the transformations shown in Figure 5B (as indicated for PCR analysis) were analyzed with optimized primers. In all 10 pale clones from transformation E (Figure 6B), the native sequence of *cpftsy* could not be amplified, indicating larger DNA insertions in all of them. The first four clones displayed the expected HR-mediated knock-in product of about 5.8 kb. When several clones carrying the same mutation had been identified, four of them were chosen for further analysis, as shown in Figure 6C (clone number and respective transformation number as indicated, compare with Figure 5B).

All clones displayed the recombinant PCR product expected after HR-mediated knock-in of HCP into *cpftsy*, and were amplified in triplets and a doublet for further analysis. After gel extraction and purification of the respective fragments, Nested-PCR was performed (Figure 6D), which showed the abundance of the inserted *aph7* gene, as well as the T2 sequence of *cpftsy* used as a control. In Figure 6E, the results of all tested clones are displayed, showing the maximal yield of specific knock-ins into *cpftsy* from transformations D and E (compare with Figure 5B), on average 35%. To further verify the specific knock-ins and provide smaller molecules for easy cloning and subsequent sequencing, PCR was again performed, as described above, using gDNA from clones 1(F) and 4(F) and partially digested thereafter with XhoI and SpeI (see Figure S2). This revealed the expected products of about 3.5 kb and 2.3 kb (XhoI), the latter digested further to 1.1 kb and 1.2 kb (SpeI) respectively. Final sequencing confirmed the HR-mediated sequence-specific knock-in in those clones, which is shown schematically in Figure 6E.

## Conclusion

Overall, the demonstrated synchronization of *C. r.* CW15 cultures is a powerful option for enhancing the efficiency of nuclear transformation, also confirmed in mammalian cells^34^ and, in particular for precise genome editing *via* HR, as demonstrated, for example, in different yeast species.^35^ Furthermore, this type of cell synchronization makes future in-depth research possible into the cell cycle of *C. r.* and could be very useful for further research into DNA damage responses^36^ during the cell cycle. The phases of the latter could be more precisely defined by using antibodies for cell cycle relevant proteins, for example, cyclins or by determining cyclin-dependent protein kinase activity.^37^ We demonstrated that access to certain cell cycle stages makes possible the genetic manipulation of the nuclear genome in different ways, depending on the desired outcome. When simple random DNA integration is sufficient, usage of the dominance of NHEJ at +4 h after illumination is the best option, since it enables the highest transformation efficiency, as also described by ref (38) for the eukaryotic parasite *Trypanosoma cruzi*.

With regard to genome editing strategies, avoiding NHEJ as much as possible and favoring HR-mediated DNA integration offers multiple benefits, including the reduction of unspecific DNA integration^39^ and enhanced gene targeting.^40^ A time point in the cell cycle of *C. r.* was identified (+12 h of illumination, Figure 4B, 6) offering a higher HR/NHEJ ratio and used to create mutants with an HR-based knock-in. Further research is required to evaluate the additional possibility of inactivating the NHEJ pathway in *C. r.*, which could reduce or even eliminate unspecific DNA integration^20,21,40^ accompanied by specific knock-ins using cell synchronization. Furthermore, the greatest efficiency of gene targeting in producing knockouts could be observed at this time point (+12 h), which confirms the advantage of HR-based gene disruption in leading to a higher probability of functional knockouts of genes. Such knockout mutants also offer the benefit of being easily identified by PCR, due to the high frequency of knock-in events using larger DNA molecules. Additional PCR analysis^33^ of targeted loci also enables the identification of the desired knock-in events (target sequence replacement by HR) that were shown to happen at acceptable rates. The actual efficiency of HR as identified in this work might be even higher, given the different outcomes of HR, as described by^41^ for the fungus *Ashbya gossypii*.

Specific knock-ins make advanced genome editing possible, for example, eliminating native genes and replacing them with altered sequences for specific purposes, such as point mutated^42^ or extended^43^ gene copies leading to tagged proteins.^44^ Thus, 5% of colonies growing on selective medium will display a desired mutant knock-in sequence even in the absence of a visible phenotype. Since *cpftsy* displays a rather inefficient target for genome editing and was mainly chosen as the optimal phenotype of a knockout, our improved strategy should increase the efficiency of genome editing for more easy accessible targets and so provide high frequencies of the creation of knockouts and specific knock-ins. Finally, valuable information concerning the nuclear HR pathway in *C. r.* could be obtained, that is, its identification in special phases (G2/M) of the cell cycle, which is consistent with other eukaryotes.^19^

## Outlook

Further optimization of HR usage could be achieved by identifying the optimal length of the homologous regions^30^ and the additional determination of optimal Cas9, sgRNA, and plasmid amounts^9^ as well as other types of DNA nucleases like Cas12a.^45^ Moreover, the strategy presented should be applicable to other algae species, when conditions of synchronization have been identified and nuclear transformation is possible,^46,47^ especially if the genetic handling of species is problematic, enabling more efficient nuclear transformation and genome editing strategies, necessary for basic research and biotechnological applications.

## Methods

### Chemicals, Reagents, and Enzymes

If not otherwise stated, all the chemicals and reagents used were provided by Sigma-Aldrich and AppliChem and all the enzymes used were supplied by New England Biolabs, Promega and Thermo Fisher Scientific.

### Gene Identity

The phytozome database (https://phytozome.jgi.doe.gov) was used for *C. r*. relevant data. Database entry for *cpftsy* is Cre05.g241450.

### Strains, Cell Culture, and Transformation

The *Chlamydomonas reinhardtii* strain CW15^7^ was used in all the experiments described and was cultivated in 20 mL flasks containing TAP-medium,^48^ supplemented with 100 μg/mL of ampicillin. Normal growth conditions were set to 25 °C with 200 μmol photons m^–2^ s^–1^ of white light for 16 and 8 h of darkness. Synchronizing growth conditions were adjusted to ref (23) with 200 photons m^–2^ s^–1^ of white light for 12 h at 28 °C, followed by 12 h of darkness at 18 °C. Cell number was determined using a Countess II FL cell counter (Life Technologies) and the results were divided by a calibration factor of 2.

### Transformation

For optimized transformation of *C. r*. CW15 based on ref (4), the respective number of cells (unsynchronized: 5 × 10^6^; synchronized: 10^6^) were harvested at 8000*g* for 7 min and resuspended in 50 μL of TOS-Medium (80% v/v TAP, 40 mM Sucrose). The suspension was mixed with different amounts of linearized plasmid DNA (pHyg3: NdeI; HCP: XbaI; for unsynchronized cells: 250 ng; for synchronized cells: in general 150 ng, 60 ng for time point +4 HCP-GE (see Figure 5A) and 400 ng for time points +8 h as well as +12 h (see Figure 5A,B). For genome editing experiments, a further 3 μg Cas9 (deviating amounts see Figure 5B) and 5.4 μg sgRNA, each consisting of 50% sgRNA for *cpftsy* target1 and target2 (deviating amounts see Figure 5B), were added and the mixture incubated on ice for 5 min. Transformation was carried out in 0.4 cm spaced cuvettes by electroporation using a Gene Pulser II (Bio-Rad) set to 200 Ω, 50 μF and 0.6 kV. Recovery was achieved in 1.5 mL TOS-Medium containing reaction tubes kept in darkness overnight on a mixing rotator. After subsequent centrifugation at 8000*g* for 7 min, cells were resuspended in 1 mL 30% starch containing TAP-Medium, supplemented with 60 μg/mL hygromycin and 100 μg/mL ampicillin. This solution was distributed on two 60 μg/mL hygromycin and 100 μg/mL ampicillin containing 1.5% Agar-TAP-plates to obtain selection.

*E. coli* strain Top10 (Thermo Fisher Scientific) and BL21 (Stratagene) were transformed with constructs using the standard heat shock method and selection was achieved by using 100 μg/mL ampicillin on 1.3% Agar-LB-plates (1% tryptone, 0.5% yeast extract and 0.5% NaCl). Subsequent cultivation was carried out at 37 °C for 16 h in LB-medium.

### Isolation of Nucleic Acids

Plasmid isolation from *E. coli* was carried out using the GeneJet Miniprep Kit (Thermo Scientific). Contrary to the supplier’s instructions, the elution buffer was prewarmed to 55 °C and elution was repeated once using the eluate.

Isolation of genomic DNA from *C. reinhardtii* CW15 was carried out after ref (49). Harvested cells (12 000*g* for 30 s) of 1–2 weeks old cultures grown in plastic multiwells of 2–3 mL TAP-Medium each were resuspended in 700 μL 2× CTAB buffer, supplemented with 100 μg Proteinase K and 50 μg RnaseA. After incubation for 2–5 h at 60 °C, genomic DNA was extracted using 1 unit of chloroform/isoamyl alcohol (24:1) after centrifugation at 12 000*g* for 5 min. This step was repeated with 1 unit of phenol (10 mM Tris-HCl buffered at pH 8)/chloroform/isoamyl alcohol (25:25:1) and 1 unit of chloroform. Precipitation was achieved using 0.3 M sodium acetate pH 5 and the addition of 1 unit of isopropanol at −20 °C for 1 h. After centrifugation at 12 000*g* for 15 min, the supernatant was removed and the sediment was washed twice with 70% ethanol at 12 000*g* for 5 min. Sediments were dried at 42 °C for 1 h and resuspended in 20–50 μL 10 mM Tris-HCL pH 8.

Isolation of *in vitro* transcribed sgRNA was carried out in the same way as described for genomic DNA, starting by increasing the final volume after the reaction to 1 mL with DEPC-H_2_O and continuing with phenol (Tris-HCl buffered pH 5)/chloroform/isoamyl alcohol (25:25:1) extraction.

Concentration of nucleic acids was determined using a Nanodrop One (Thermo Scientific). In the case of plasmid DNA for transformation of *C. reinhardtii*, concentration was precisely determined on 1% agarose gels compared to the marker GeneRuler 1kb Plus (Thermo Scientific) using the ImageJ software (https://imagej.nih.gov/ij/).

### Amplification, *In Vitro* Transcription, Restriction, and Cloning

Taq polymerase was expressed in *E. coli* Bl21 and isolated according to the literature.^50^ As a general buffer, 10 mM Tris-HCl pH 8.3, 50 mM KCl and 4 mM MgCl_2_ were chosen, while special amplifications based on GC rich templates like those present in *cpftsy* (5′ Flanking region, see Figure 3) were performed using 75 mM Tris-HCl pH 8.3, 20 mM (NH_4_)_2_SO_4_ and 4 mM MgCl_2_. Reactions also contained 10 pmol of each primer, 0.53 mM dNTPs (each) and 0.26 M Betain. Annealing temperatures 2–5 degrees below the supplier’s reported melting temperature of primers were chosen, with elongation to 1 min/kb at 72 °C. All amplifications for cloning were achieved by using Hybrid Polymerase (EURX) in accordance with the supplier’s instructions. In addition, target sequence analysis of *cpftsy* using flanking regions surrounding primers (see Figure 3) was carried out using the same buffer, containing (NH_4_)_2_SO_4,_ as that for Taq amplifications of GC rich templates.

Amplification of template DNA for *in vitro* transcription of sgRNA was obtained using Taq polymerase with the NH_4_ buffer, as mentioned above, using primers (see Table S2) sg_CP_fw1 and T7_CP_fw1 (for target T1, see Figure 3) as well as sg_CP_fw2 and T7_CP_fw2 (for target T2, designed using www.e-crisp.org, see Figure 3). As a first step, target sequence (5′) elongated primers binding the Cas9 required RNA sequence (sg_CP_fw1, sg_CP_fw2) on plasmid pDGE277^51^ were used. After dilution of the PCR products obtained to about 0.1–1 ng/μL, a second amplification was performed with primers binding the target sequence and containing a 5′ addition of the T7 polymerase motif (T7_CP_fw1, T7_CP_fw2). After determining the concentration of the PCR products obtained, 1 μg of template DNA was used for *in vitro* transcription, using the Hi Scribe T7 Quick High Yield RNA Synthesis Kit (New England Biolabs) at 37 °C overnight, with subsequent purification of sgRNA as mentioned above. Recombinant Cas9 activity was tested at 37 °C for 1 h using 100 ng of PCR product created with Primers Cp_fw and Cp_rv (see Figure 3), 400 ng sgRNA for *cpftsy* target 1 and 2 (see Figure 3), and 750 ng of recombinant Cas9 (see chapter below) in cleavage buffer (20 mM Tris-HCl pH 7.5, 20 mM KCl, 5 mM MgCl_2_).

Plasmid DNA was restricted using the enzymes XhoI, SpeI, NdeI, XbaI, NheI, and EcoRV at 37 °C for 3–16 h, with subsequent heat inactivation at 65 °C for 10 min. Isolation of PCR products for cloning was achieved using the GeneJET Gel Extraction Kit (Thermo Scientific) and initially cloned into Blueskript KS- (Snapgene) for sequencing (Eurofins), subsequent restriction and ligation using T4 Ligase into final constructs.

### Expression and Purification of Recombinant Cas9

*E. coli* strain BL21 was transformed with the Cas9 nuclease (160 kDa) encoding plasmid pET- NLS-Cas9-6xHis (Addgene). After expression, the recombinant Cas9 contained a nuclear localization signal and a C-terminal his-tag for purification using nickel affinity chromatography. After overnight cultivation of a 5 mL culture containing 25 μg/mL chloramphenicol and 100 μg/mL ampicillin, 1 L LB-Medium containing the same antibiotics was inoculated and the culture grown to an OD_600_ of 0.5. Expression of recombinant Cas9 was induced with 0.5 mM isopropyl β-d-1-thiogalactopyranoside, and cultivation continued overnight at 20 °C. Cells were harvested at 5000*g* for 10 min and purified in accordance with ref (52) using Nickel NTA affinity chromatography. In order to remove nucleic acids from *E. coli* from the protein solution, the latter was supplemented with 10 mM EDTA and incubated for 1 h. Subsequent concentration using centricons (10 kDa cutoff) was carried out twice to a small volume and again diluted in order to remove nucleic acids and EDTA, achieving a final volume of 750 μL. Thereafter, the protein solution was analyzed on SDS-page^53^ and immunoblotting was carried out. Protein concentration was determined using a Nanodrop One with an extinction coefficient at 280_nm_ of 120.450 M^–1^ cm^–1^.

### Construction of Knock-In Construct HCP

All the elements necessary for creating HCP (5′ flanking (5F): chromosome_5:3459480–3461402, *aph7* cassette, 3′ flanking (3F): chromosome_5:3461425–3463347; see Figure 3) were amplified as described above using 5F_fw/rv (5F), pBT_fw/rbsc2_rv (*aph7* cassette) and 3F_fw/rv (sequences see Table S2), cloned into bluescript vector KS- and sequenced. The 5′ flanking region (5F) was amplified using a reverse primer (5F_rv) that also carried nucleotides for an in-frame stop codon after sequence-specific knock-in into *cpftsy*. The *aph7* cassette from the transformation vector pHyg3,^54^ conferring resistance to hygromycin, consisted of the promoter from the β-tubulin gene, the *aph7* gene itself and the 3′ UTR of the RubisCO small subunit 2 gene. Further sequence extension of used primers provided restriction motifs for *Eco*RI and XbaI (5F) as well as NheI and XbaI (*aph7* cassette and 3F). All elements were cloned again into bluescript KS- in the order shown (Figure 3), leading to the HCP construct.

## Supporting Information Available

Figure S1: Recombinant Cas9 purification and activity verification; Figure S2: Amplification and restriction analysis of *cpftsy* fragments after HR mediated knock-in; Table S1: Overview of transformation procedure of *C. r.*; Table S2: Sequence of used oligonucleotides (PDF)
