# Supplementary material for: Cell Synchronization Enhances Nuclear Transformation and Genome Editing via Cas9 Enabling Homologous Recombination in Chlamydomonas reinhardtii
Source: ACS Synth Biol. 2020 Sep 11;9(10):2840–50. doi: 10.1021/acssynbio.0c00390 (PMC8011982; doi:10.1021/acssynbio.0c00390)
Supplement: Supplementary file 1 — sb0c00390_si_001.pdf [file sb0c00390_si_001.pdf]

Supporting Information

Title: Cell synchronization enhances nuclear transformation and genome editing via Cas9 enabling Homologous Recombination in *Chlamydomonas reinhardtii*

Authors: Max Angstenberger, Francesco de Signori, Valeria Vecchi, Luca Dall'Osto and Roberto Bassi

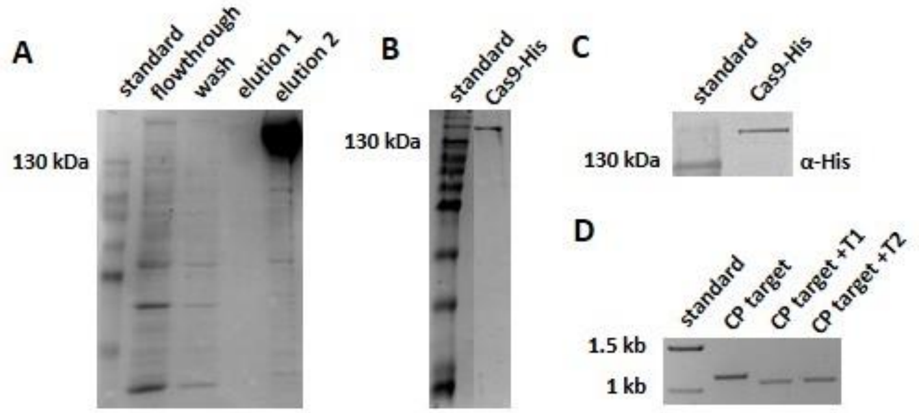

Figure S1. Result of expression and purification of nuclease Cas9 from *E. coli*. (A) SDS-PAGE analysis after expression and purification of recombinant Cas9-His in *E. coli*, carried out on eluted fractions from Ni-affinity chromatography. (B) SDS-PAGE analysis of Cas9-His after EDTA treatment, washing and concentration by centricons. (C) Immunoblot using an α-His antibody for detection of Cas9-His. (D) *In vitro* restriction assay using Cas9-His, amplified and purified target DNA of *cpftsy* (CP target, see Fig. 3: CP_fw/CP_rv) and sgRNA for target T1 and T2.

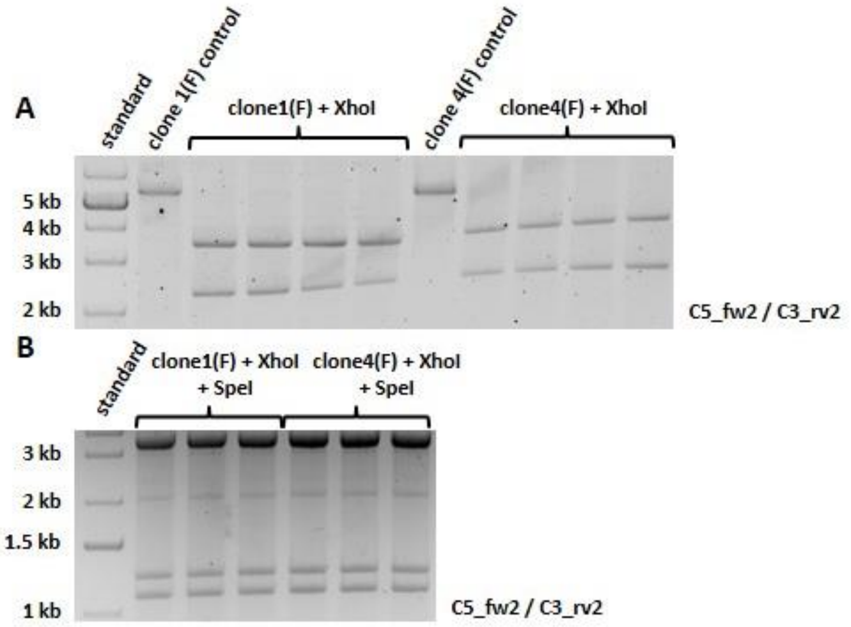

Fig. S2 Agarose gels showing restriction analysis of *cpftsy* knock-in amplification products. After amplification of the recombinant *cpftsy* sequence containing the desired knock-in of the *aph7* cassette using the primers C5_fw2 and C3_rv2 (see Fig. 3, 6), in one reaction for clones 1F and 4F (A), one part was loaded as a control (5.8 kb), while the rest was digested with XhoI and loaded onto 4 lanes, leading to the restricted fragments of 2.3 kb and 3.5 kb as expected. In a second reaction, also using SpeI (Fig. 6), the fragment of 2.3 kb was further digested into one fragment of 1.1 kb and another of 1.2 kb. Fragments of 3.5 kb, 1.2 kb and 1.1 kb were purified by gel extraction and cloned into the bluescript vector KS- for sequencing, which confirmed the occurrence of sequence-specific knock-in, as shown in Fig. 6F.

Table S1 Transformation procedure overview. Shown are the original and optimized transformation protocols for *C. r.* CW15 used in this study, showing an overall optimization and an especially marked reduction of the necessary compounds due to the use of synchronized cell cultures with a very high transformation efficiency.

	**Original protocol unsynchronized cells [based on Shimogawara *et al.*, 1998[4]]**	**Optimized protocol unsynchronized cells**	**Optimized protocol synchronized cells**
**Light / Temperature**	24 °C; 16 h light, 8 h darkness	24 °C; 16 h light, 8 h darkness	28°C 12 h light; 18 °C 12 h darkness
**Doubling time**	8 - 10 h	8 - 10 h	24h
**Transformation culture**	0.5 x 10^6 cells/ml; 3-4 days	10^5 cells/ml; 3 days	10^5 cells/ml; 3 days
**Cells / transformation**	5 x 10^7	5 x 10^6	10^6
**Linearized DNA**	1 µg	250 ng	60 – 400 ng
**Transformation volume**	250 µl	50 µl	50 µl
**Recovery volume**	20 ml	1.5 ml	1.5 ml
**Plates / Transformation**	6	2	2
**Cas9**	5 µg – 15 µg	3 µg	3 µg – 6 µg
**sgRNA**	8 – 12 µg	5.4 µg	5.4 µg – 40 µg
**Timepoint of transformation [hours after illumination start]**	+4	+4	0, +1, +2, +4, +6, +8, +10, +12
**Hygromycin [µg/ml]**	25	60	60

Table S2 Primers used in this study.

Primer	Sequence (5‘ -> 3‘)
CP_fw	GTCACCTCGAATCACACAC
CP_fw2	GAAGGAGTGAGATGACTTGGAC
CP_rv	CTCCTCCACCTCTTCTGCTC
C5_fw	TGGAGATCCAGGTCGGTTGC
C5_fw2	TTGTAAAGTGAGGTCCAGAGGAGC
C3_rv	CCAGGTTCTGTTGGTTGGC
C3_rv2	TGCCGTGTCTACCAGAATGAGGTC
5F_fw	GCGAATTCCGCTTGAGGAACACATAC
5F_rv	GCTCTAGATTAGCCGAAGTCGGCGGACTG
pBT_fw	CTATGCTAGCCTTGCGCTATGACACTTC
Aph7_fw	ATGCGCTAGCATGACACAAGAATCCCTG
Aph7_rv	TCTAGATTATCAGGCGCCGGGGGCGG
rbsc2_rv	GCTCTAGACGCTTCAAATACGCCCAG
3F_fw	TATAGCTAGCACCGCATCCGCGAGGG
3F_rv	CGTCTAGAGTGGTAGTTGGTGTACG
Cao_fw	TGTTACCGCATAGAGCAGCC
Cao_rv	CGCTACTCACCGCTGAGAAC
sg_CP_fw1	CGATCTTCAGAGCAGTGCGGGTTTTAGAGCTAGAAATAGCAAG
T7_CP_fw1	TAATACGACTCACTATAGCGATCTTCAGAGCAGTGCG
sg_CP_fw2	GATCGCGTACAAGTACGGCAGTTTTAGAGCTAGAAATAGCAAG
T7_CP_fw2	TAATACGACTCACTATAGGATCGCGTACAAGTACGGCA
Cas9_rv	AAAAAAAGCACCGACTCGGTGCCAC
